# Difunctional Hydrogel Optical Fiber Fluorescence Sensor for Continuous and Simultaneous Monitoring of Glucose and pH

**DOI:** 10.3390/bios13020287

**Published:** 2023-02-17

**Authors:** Yangjie Li, Site Luo, Yongqiang Gui, Xin Wang, Ziyuan Tian, Haihu Yu

**Affiliations:** National Engineering Research Center of Fiber Optic Sensing Technology and Networks, Wuhan University of Technology, Wuhan 430070, China

**Keywords:** hydrogel fiber, glucose, pH, fluorescence sensor, PBA, fluorescein derivative

## Abstract

It is significant for people with diabetes to know their body’s real-time glucose level, which can guide the diagnosis and treatment. Therefore, it is necessary to research continuous glucose monitoring (CGM) as it gives us real-time information about our health condition and its dynamic changes. Here, we report a novel hydrogel optical fiber fluorescence sensor segmentally functionalized with fluorescein derivative and CdTe QDs/3-APBA, which can continuously monitor pH and glucose simultaneously. In the glucose detection section, the complexation of PBA and glucose will expand the local hydrogel and decrease the fluorescence of the quantum dots. The fluorescence can be transmitted to the detector by the hydrogel optical fiber in real time. As the complexation reaction and the swelling–deswelling of the hydrogel are all reversible, the dynamic change of glucose concentration can be monitored. For pH detection, the fluorescein attached to another segment of the hydrogel exhibits different protolytic forms when pH changes and the fluorescence changes correspondingly. The significance of pH detection is compensation for pH errors in glucose detection because the reaction between PBA and glucose is sensitive to pH. The emission peaks of the two detection units are 517 nm and 594 nm, respectively, so there is no signal interference between them. The sensor can continuously monitor glucose in 0–20 mM and pH in 5.4–7.8. The advantages of this sensor are multi-parameter simultaneous detection, transmission-detection integration, real-time dynamic detection, and good biocompatibility.

## 1. Introduction

Diabetes caused by a glucose metabolism disorder afflicts millions of people worldwide [1,2]. Early detection and treatment of diabetes mellitus is of great significance in reducing the long-term harm caused by diabetes mellitus and its complications [3]. Continuous glucose monitoring (CGM) is a feasible method for real-time and dynamic detection of glucose concentration by implanting glucose sensors into the subcutaneous tissue, which can be used in combination with an insulin injection pump to help people with diabetes with treatment [4]. Continuous glucose monitoring means that the output signal of the sensor changes with the glucose concentration, and the patients can acquire information about their glucose level at any time without frequent sampling [5]. Today, continuous glucose monitoring in the clinic is achieved by measuring enzyme reaction electricity while the enzyme electrode is implanted in subcutaneous tissue [6,7]. However, this method requires three to four fingersticks per day to calibrate the sensor, which can cause discomfort to patients [8]. Additionally, the electrochemical sensor has signal drift due to the instability of electrochemical reactions in vivo, which reduces the accuracy of glucose detection. In addition, the pH value is also a crucial physiological parameter [9,10]. Each area of cells is exposed to different acidity levels, and only an appropriate pH level can maintain its normal biological function [11]. For example, the pH value of blood is 7.35–7.45 [12]. However, when acid–base homeostasis is not balanced, for example, in acidosis, interstitial pH can decrease below 7.35; in alkalosis, the pH of the interstitial fluid may exceed 7.45. Thus, it is necessary to detect the pH value as well as glucose concentration [13,14].

As promising detection platforms for continuous glucose and pH monitoring, optical glucose and pH sensors attract a lot of attention [9,15]. Optical sensors have the advantages of label-free detection, long-term continuous glucose monitoring, and electromagnetic interference resistance over electrochemical assays [16]. Various optical glucose and pH sensors have been reported recently, including fluorescent sensors [11,17,18], surface plasmons resonance (SPR) sensors [19,20,21], and surface-enhanced Raman scattering (SERS) spectroscopy sensors [22]. Among them, fluorescent sensors have been given a lot of attention in biosensing because of their biocompatibility, excellent optical properties, high selectivity, and low cost [23,24]. As representative fluorophores, quantum dots and fluorescein have been reported in glucose and pH detection [25,26,27]. However, cuvette-based fluorescence sensing limits their further application for in situ detection of pH and glucose concentration, and frequent sampling is not only a severe waste but also causes discomfort to patients. How to improve the fluorescence sensor based on the cuvette method into an implantable sensor that can realize in situ detection is a challenge for us. Combining hydrogel optical fiber with fluorescence detection is a promising approach, as fluorophores can be immobilized on the hydrogel optical fiber and further applied in implantation and continuous monitoring [28,29,30,31]. 

Hydrogels have attracted more and more attention in biosensing because of their good biocompatibility [32], excellent mechanical properties [33,34], tunable optical propertie s [35], and easy chemical modification. Silica optical fibers have been widely used in biochemical sensing [36,37,38,39]. Zhang et al. proposed an LSPR-based optical fiber biosensor for creatinine detection. The sensitivity of the proposed sensor is 0.0025 nm/μM with a limit of detection of 128.4 μM over a linear detection range of 0–2000 μM [40]. As a novel application form, hydrogel-based optical fibers with a core-cladding structure have a broad prospect in continuous glucose monitoring, which possesses both stable optic transmission and biocompatibility over silica optical fibers [34,35,41]. Meanwhile, hydrophilia and the porous network structure of the hydrogel fiber allow small molecules and ions, such as glucose and protons, to diffuse into the hydrogel fiber core, which enables optical chemical sensing in the hydrogel fiber [28]. When fluorophores are immobilized on the hydrogel fiber core, fluorescence detection can be realized in the hydrogel fiber, and the fluorescence can be transmitted to the spectrometer. Zhao et al. fabricated a flexible hydrogel optical fiber, which consisted of a permeable core/clad structure with a step-index profile for highly efficient excitation light guiding and fluorescence emission collecting [30]. The prepared fluorescent sensor displayed linear responses to pH values ranging from 3.79 to 9.55 with a high sensitivity of 0.68 per pH unit. However, leakage may occur in this sensing system because the fluorescent dye is not covalently immobilized on the hydrogel fiber.

For glucose detection, PBA is an excellent choice for non-enzyme glucose sensing because of its high affinity to carbohydrates and reversible reaction with carbohydrates [42]. However, glucose sensors utilizing PBA as the glucose-sensitive chelating agent are sensitive to pH [43]. Given the problem, coupling a pH sensor into the glucose sensing system is feasible to eliminate the measurement errors caused by pH fluctuation on glucose detection, thereby improving the detection accuracy and anti-interference of the sensor [44]. Thus, a two-parameter sensor may be established to monitor glucose and pH simultaneously by coupling a pH sensor into the PBA-based glucose sensor. Integrating multi-parameter sensing into one biocompatible sensor is of great significance for the implantation and miniaturization of biosensors [45]. There are many reports about glucose and simultaneous pH detection, but most can not realize continuous pH and glucose monitoring [46,47,48]. Jankowska et al. reported a fluorescent sensing system that could realize simultaneous detection of the pH value and glucose concentrations for wound monitoring applications [49]. Nevertheless, glucose oxidase (GOx) is prone to inactivation, which makes the sensing system unstable.

Here, we developed a hydrogel optical fiber fluorescence sensor that coupled two sensitive agents into one hydrogel optical fiber (functionalized fluorescent hydrogel optical fibers) for simultaneous continuous glucose and pH monitoring. The hydrogel optical fiber had a poly (acrylamide-co-poly (ethylene glycol) diacrylate) p(AM-co-PEGDA) core and a Ca alginate cladding. The pH-sensitive agent was covalently incorporated into the different segments of the same hydrogel core as the glucose-sensitive agent. The glucose recognition agent (3-(acrylamide)-phenylboronic acid) cross-linked with acrylamide in a part of the hydrogel core. Moreover, the 3-APBA functionalized fiber part was doped with quantum dots as the signal converter, which converted the hydrogel swelling into a fluorescent signal attenuation. As the glucose complexed with the boronic acid groups immobilized on the hydrogel core, the Donnan osmotic pressure increased. Variation in the osmotic pressure causes the change in density and refractive index of the hydrogel fiber, which further reduces the fluorescence of the quantum dots. Thus, the glucose concentration can be quantified by measuring the fluorescence of the quantum dots. As the glucose-PBA reaction is pH sensitive, the fluorescein derivative was covalently incorporated into another part of the hydrogel fiber core for pH detection to compensate for the pH errors. The fluorescein exhibits different protolytic forms when pH changes and the fluorescence changes correspondingly. The Fluorescence of the quantum dots and the fluorescein derivative is transmitted to the spectrometer through the hydrogel core and an embedded multimode fiber. Glucose concentration and pH can be detected simultaneously as there is no fluorescence overlap between fluorescence emission (517 nm) and quantum dots emission (600 nm). The sensor can continuously monitor glucose and pH as two reactions of the sensor are reversible. The biocompatible and flexible hydrogel optical fiber has implantation potential. 

## 2. Experimental

### 2.1. Reagents and Instruments

CaCl_2_, D-glucose, D-fructose and sodium lactate, and sodium borohydride (NaBH_4_) were obtained from Sinopharm Chemical Reagent Co. Reduced glutathione (GSH), tellurium powder, cadmium chloride hydrate (CdCl_2_•2.5H_2_O), N-Hydroxysuccinimide (NHS), N-(3-Dimethylaminopropyl)-N’-ethylcarbodiimide hydrochloride (EDC), 1,2,4-benzenetricarboxylic acid, methanesulfonic acid, resorcinol, N, N-Dimethylformamide, poly(ethylene glycol) diacrylate (PEGDA, 600 DA), 2-hydroxy-2-methylpropiophenone (2-HMP), N-(3-Aminopropyl) methacrylamide hydrochloride, acrylamide (AM), and 3-(acrylamido) phenylboronic acid (3-APBA) were purchased from Aladdin Reagent Co., Ltd. (Shanghai, China). Phosphate buffered saline (0.1 M) was prepared by disodium hydrogen phosphate (Na_2_HPO_4_) and sodium dihydrogen phosphate (NaH_2_PO_4_). All the reagents were analytical grade and used without further purification, and the water used in the experiments was ultrapure water produced with a Hitech-K flow water purification system. 

Emission spectra of the fluorophore were recorded by an F-4500 FL Spectrophotometer (Hitachi, Tokyo, Japan). The absorption spectra were measured by a UV-2450 UV–visible spectrophotometer (Shimadzu Co., Ltd., Tokyo, Japan). Fourier transform infrared spectra (FT-IR) were collected with a Nexus spectrometer. The transmission electron microscopy (TEM) images were observed by the JEM-2100F (JEOL Ltd., Tokyo, Japan). Dynamic light scattering (DLS) and Zeta potential were measured by a Zetasizer Nano ZS. SC light source (450–2400 nm, SC-COMPACT, OYSL Co., LTD, Wuhan, China) and QE65000 spectrometer (Ocean Optics, Dunedin, FL, USA) were applied for the optical sensing platform.

### 2.2. Synthesis of CdTe QDs

CdTe QDs were synthesized using previously reported methods with some modifications [50,51]. In brief, the deionized water was first deoxygenated under a nitrogen atmosphere before synthesis, since the synthesis needs to be carried out in an anaerobic environment. Then, 31.9 mg of Te powder and 50 mg of NaBH_4_ were added to the deoxygenated deionized water and stirred in a sealed flask. The suspension gradually became a light purple solution and finally a colorless NaTeH4 solution. Meanwhile, 0.1142 mg of CdCl_2_•2.5H_2_O and 0.3837 mg of GSH were dissolved in the deoxygenated deionized water (120 mL) to prepare the cadmium precursor, and pH was adjusted to 9 with 1.0 M NaOH. The cadmium precursor was stirred for 30 min in a sealed flask. The color of the solution changed from colorless to purple when NaTeH_4_ solution was poured into the cadmium precursor. The solution was stirred under RT for 20 min and refluxed (99 °C) for 80 min later. Finally, the QDs were precipitated with acetone and purified by centrifugation. The purified CdTe QDs were dispersed in ultrapure water and stocked for further use.

### 2.3. Synthesis of a Fluorescein Derivative

The synthesis process scheme was exhibited in Scheme S1. A total of 2.1 g of 1, 2, 4-benzene tricarboxylic acid, 10 mL of methanesulfonic acid, and 2.2 g of resorcinol were added to a flask and heated at 90 °C for 72 h. After cooling to room temperature, the dark brown solution was poured into 150 mL of an ice/water slurry and stirred vigorously to precipitate an orange-red solid. The solid was dried in the air and used directly without further purification. A total of 254 mg of N-(3-Dimethylaminopropyl)-3-ethyl carbodiimide hydrochloride (EDC•HCl) and 155 mg of N-hydroxysuccinimide (NHS) were dissolved in dry DMF (4 mL) in a flask. The prepared solid (1 g) in dry DMF (2 mL) was added dropwise to the above EDC/NHS solution. The reaction was stirred at room temperature for 48 h in the dark. The resulting bright yellow solution contained fluorescein-NHS, which was used directly next.

### 2.4. Fabrication of Difunctional Fluorescent Hydrogel Optical Fiber

**Figure 1:**
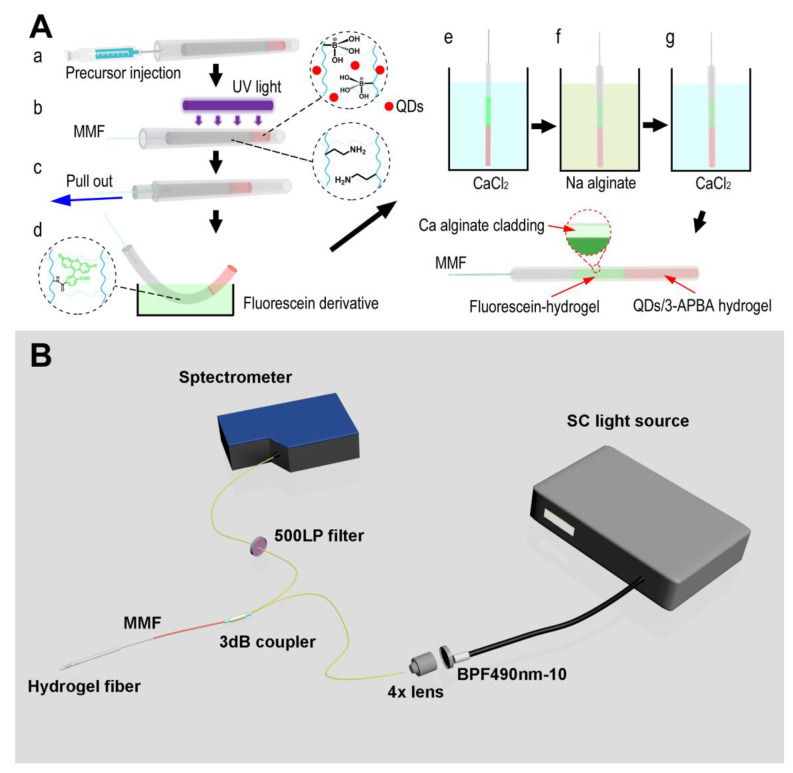
(**A**) Fabrication of difunctional fluorescent hydrogel fiber. (**B**) Optical setup for simultaneous continuous pH and glucose monitoring.

The blank hydrogel fiber was fabricated by following a previous method with a minor modification, as Figure 1A shows [41]. The precursor solution containing AM and PEGDA (85:15 mol%) was injected into a silicone tube mold (Cole-Parmer), and the UV light was utilized to initiate cross-linking. Then, 5 μL 2-HMP was added as the initiator. The hydrogel optical fiber core was pulled out from the tube after the tube swelled in the dichloromethane. Soon afterward, the core was immersed in CaCl_2_ (0.1 M) solution for 30 min. Then, the core was dipped in the Na alginate solution (1 wt%) to form a Ca alginate hydrogel cladding. At last, the fabricated pre-hydrogel fiber was immersed in the CaCl_2_ (0.1 M) solution for further cross-linking of alginate chains.

For the fabrication of difunctional hydrogel optical fiber, two precursors were prepared. The precursors containing QDs/3-APBA (AM:PEGDA:3-APBA = 82:3:15 mol%) and N-(3-Aminopropyl) methacrylamide hydrochloride:AM:PEGDA = 1:85:14 mol%) were prepared. A total of 5 μL 2-HMP was added to the precursors. The two precursors were successively injected into a silicone tube mold, and a blank precursor (AM: PEGDA = 85: 15 mol%) was injected into the tube last. The silicone tube containing precursors was exposed to UV light (365 nm). A multimode fiber was preset in the blank precursor before cross-linking (Scheme S2 and Scheme S3). The QDs/3-APBA was set at the distal end to avoid the effects of the hydrogel expansion on fluorescein fluorescence. Soon afterward, the amino hydrogel part was immersed in the fluorescein derivative solution (pH 7.4) (10,000 × dilutions) for 6 h and then immersed in KCl (0.1 M) to remove the electrostatic adsorbed N-(3-Aminopropyl) methacrylamide hydrochloride (Scheme S4). The following processes were the same as mentioned above.

### 2.5. Experimental Setup

Figure 1B shows the optical setup of the sensing system. The 490 nm light was obtained from the SC light source configured with a BPF490 nm-10. The light was coupled into the hydrogel through a 3 dB coupler, and the MMF was embedded in the hydrogel fiber. When the fluorophores were excited, fluorescence was transmitted back to the spectrometer. A long-pass filter with a cut-off wavelength of 500 nm was used to remove the excitation laser.

## 3. Results and Discussions

### 3.1. Fabrication of Fluorophores and Difunctional Fluorescent Hydrogel Optical Fibers and Their Characteristics

**Figure 2:**
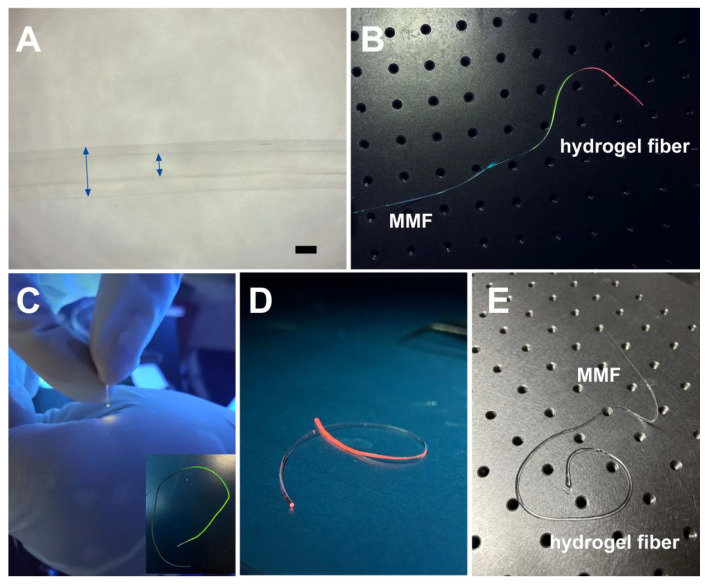
The photographs of the fluorescent hydrogel fiber. (**A**) The optical microscope photograph of the hydrogel fiber. Scale bar = 0.5 mm. (**B**) The fluorescence photograph of fluorescent hydrogel fiber excited with 490 nm light. (**C**,**D**) Fluorescence photographs of hydrogel fiber underside UV light (365 nm) illumination. (**E**) Photographs of the fabricated hydrogel fibers.

**Figure 3:**
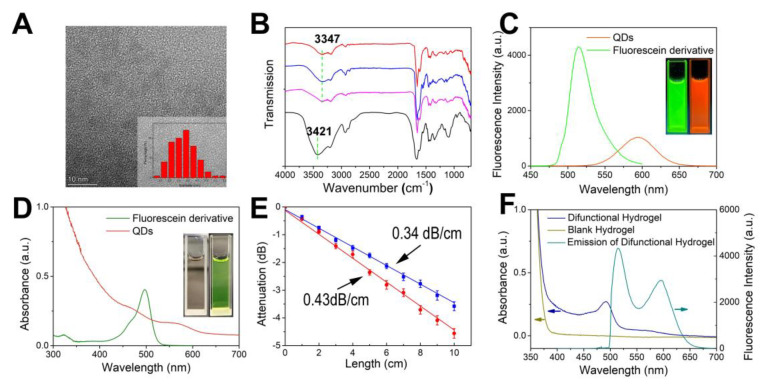
(**A**) TEM image and size distribution of CdTe QDs. (**B**) FT-IR. Top to bottom: blank hydrogel, amino hydrogel, fluorescein derivative functionalized hydrogel, and 3−APBA functionalized hydrogel. (**C**) Emission spectra of fluorescein derivative and QDs. Insets: fluorescence photographs of fluorescein derivative and QDs under UV light (365 nm) illumination. (**D**) Absorption spectra of fluorescein derivative and QDs. Insets: solution under white light. (**E**) The propagation loss of hydrogel fibers in the air with/without cladding, measured by a cutback technique. The wavelength of the light was 532 nm. (**F**) Absorption spectra of blank hydrogel and difunctional hydrogel. Dual emission was acquired from the spectrometer of the monitoring system.

The precursor solution containing AM and PEGDA (85:15 mol%) was injected into a silicone tube mold, and the UV light was utilized to initiate cross-linking. Then, the hydrogel fiber core was pulled out from the tube after the tube swelled in the dichloromethane. Then, Ca alginate cross-linked on the surface of the hydrogel core in CaCl_2_ (0.1 M) solution to form the cladding. Ca alginate could improve the mechanical properties of hydrogel optical fibers as well. Figure 2E exhibited photographs of the hydrogel optical fiber. A distinct core cladding is observed under the microscope, and the diameter is 0.61 mm/1.1 mm (Figure 2A). The diameter of the hydrogel fiber is tunable. The absorbance spectrum indicated the transmission of the hydrogel (Figure 3F). High transmission in a visible light band is beneficial for fluorescence sensing. The optical loss of hydrogel optical fibers increased exponentially (linearly in dB scale) with the fiber length (Figure 3E). Furthermore, the measured loss of core-only fibers was 0.43 dB/cm, which was greater than the core/clad hydrogel fiber. In addition, the mechanical properties of the fiber were measured. The hydrogel fiber showed a Young’s modulus of about 22 MPa (Figure S1, in the Supplementary Material).

The CdTe QDs were fabricated by a hydrothermal method. The TEM photograph (Figure 3A) indicated the QDs were monodispersed, and the average diameter was ~3.5 nm. The DLS diameter of the CdTe QDs is ~6.1 nm. The fluorescein derivative was synthesized through a series of reactions (Scheme S1). The hydrogel optical fiber segmentally functionalized by the fluorescein derivative and quantum dots/3-APBA (difunctional fluorescent hydrogel optical fiber) was fabricated by successively injecting two precursors into a silicone tube mold and cross-linking under UV light. A multimode optical fiber was preset in the precursor before cross-linking. The following processes were the same as mentioned above. The 3-APBA functionalized hydrogel part was synthesized by cross-linking the PBA derivative with AM and PEGDA. The cross-link was verified by FT-IR. As Figure 3B shows, the 3-APBA functionalized hydrogel had a broad peak at 3421 cm^−1^ compared to blank hydrogel, which is attributed to O-H stretching vibrations [52]. The broad peak comes from the boric acid of 3-APBA. The QDs were doped into the PBA functionalized hydrogel part by mixing the QDs with the PBA-containing precursor before cross-linking. The QDs could be immobilized on the PEGDA hydrogel [53]. N-(3-Aminopropyl) methacrylamide hydrochloride cross-linked with AM and PEGDA to form the amino hydrogel. As Figure 3B showed, the broad peak at 3347 cm^−1^ (N-H stretching vibrations) compared with the blank hydrogel indicated that N-(3-Aminopropyl) methacrylamide hydrochloride was introduced into the hydrogel. The amino hydrogel fiber was immersed in the fluorescein derivative solution (pH 7.4) for 6 h. The fluorescein derivative reacted with the amino on the hydrogel to form the amide, thereby being covalently immobilized on the hydrogel. The FT-IR affirmed this, the broad peak of the amino hydrogel disappeared and recovered to the same as the blank hydrogel after reaction with the fluorescein derivative. It was worth noting that a part of the unreacted fluorescein derivative would be absorbed into the amino hydrogel due to electrostatic adsorption. Zeta potentials affirmed this as the zeta potentials of N-(3-Aminopropyl) methacrylamide hydrochloride and fluorescein derivative were 27.2 ± 1.1 mV and −11 ± 0.6 mV, respectively. High ionic strength solution could desorb the absorbed fluorescein derivative [54]. The electrostatically adsorbed fluorescein derivative was removed by immersing the hydrogel fiber in the KCl solution (0.1 M) to avoid leakage in subsequent use.

The absorption and emission spectra of QDs and fluorescein derivatives in water were measured. Two shoulder peaks of the QDs were at 475 nm and 580 nm (Figure 3D). Under 490 nm excitation, QDs had an emission peak at 594 nm. The QDs can be excited in a broad range of excitation from 460 nm to 500 nm, as Figure S2 shows. The fluorescein derivative had a dual-hump-like absorption peak at 450 nm and 490 nm, which was related to pH as its protolytic equilibrium involved four species [55]. The absorption spectra in various pH was shown in Figure S3. The fluorescein derivative is also excitation independent as it can be excited in a range of excitation from 390 nm to 490 nm (Figure S4). It has a 517 nm emission under 490 nm excitation. Bright green and bright red fluorescence were observed when the QDs and fluorescein derivative were under UV light irradiation (Figure 3C insets). The difunctional fluorescent hydrogel optical fiber had similar absorption and emission properties to fluorophores in water (Figure 3F). It had two absorption peaks at 475 nm and 490 nm corresponding to the QDs and fluorescein derivative, respectively. A dual emission was found when 490 nm light was coupled into the difunctional fluorescent hydrogel optical fiber through a multimode fiber embedded in the hydrogel to excite the QDs and the fluorescein derivative (Figure 3F). Red and green fluorescence at the functionalized area were observed from the side of the hydrogel optical fiber under 490 nm light excitation (Figure 2B). We also irradiated the functional area with UV light from the side of the hydrogel optical fiber. Except for the side fluorescence, we could also observe the strong fluorescence from the end face of the hydrogel optical fiber (Figure 2C,D). The fluorescence was guided through the hydrogel optical fiber and projected onto a screen, as the photograph showed. This means that the fluorescent hydrogel optical fiber sensor may lower the light source requirement. A UV light may be enough for excitation rather than an expensive light source. However, calibration is a foreseeable problem.

### 3.2. Difunctional Fluorescent Hydrogel Optical Fiber for pH Monitoring

The mechanism of pH detection is discussed as follows. We have introduced the pH sensitivity of the fluorescein derivative in brief. As pH increased from 3.4 to 7.8, the absorption peak of fluorescein derivative at 490 nm enhanced. Emission peak at 517 nm under 490 nm excitation enhanced correspondingly. As pH changes, the interconversion of four protolytic forms leads to changes in the fluorescein derivative’s absorption and emission. The four forms are the dianion (F^2−^), the monoanion (FH^−^), the neutral (FH_2_) form, and the cation (FH_3_^+^) so that three acid-base equilibria are expected as shown below.

$${\mathrm{FH}}_{3}^{+}+H_{2}O\underset{}{⇄}{\mathrm{FH}}_{2}{+H}_{3}O^{+}\;\;{\mathrm{pKa}}_{1}$$

$${\mathrm{FH}}_{2}{+H}_{2}O\underset{}{⇄}{\mathrm{FH}}^{-}{+H}_{3}O^{+}\;\;{\mathrm{pKa}}_{2}$$

$${\mathrm{FH}}^{-}{+H}_{2}O\underset{}{⇄}F^{2}{}^{-}{+H}_{3}O^{+}\;\;{\mathrm{pKa}}_{3}$$

**Figure 4:**
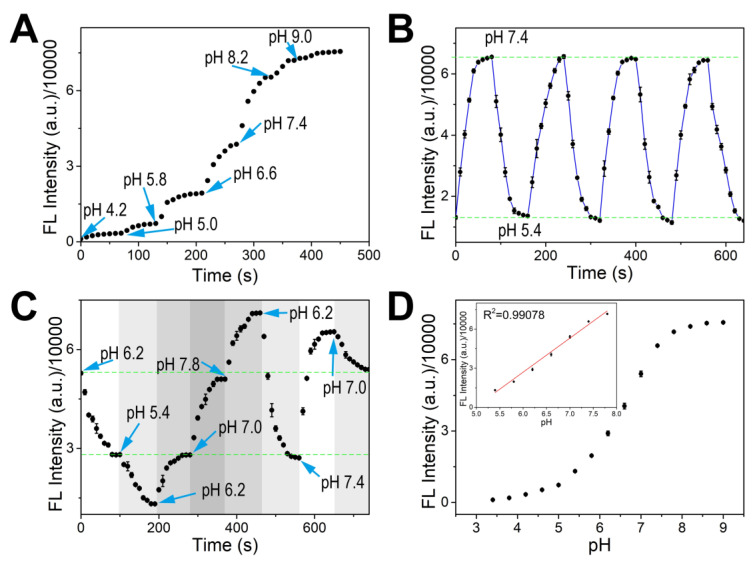
(**A**) Time response of pH detection from pH 3.4 to pH 9.0. The dots represent the fluorescence intensity recorded every 10 s, which is the same as (**B**,**C**). The moments when pH starts to change are pointed out with arrows, which is the same as (**C**). (**B**) Repeatability of the sensor for pH detection from pH 5.4 to pH 7.4. The green dashed lines represent reference fluorescence intensity corresponding to pH 7.4 (up) and pH 5.4 (down). (**C**) Time response of the sensor for continuous pH monitoring (pH 7.0→6.2→5.4→6.2→7→7.8→6.2→7.4→7.0). The green dashed lines represent reference fluorescence intensity corresponding to pH 6.2 (down) and pH 7.0 (up). (**D**) Detection curve of the sensor for pH. Inset: linear relation of fluorescence intensity to pH. Error bars in (**B**–**D**) are based on standard deviations (n = 3).

The fluorescence and absorption properties of the fluorescein derivative immobilized on the hydrogel optical fiber are similar to the free fluorescein derivative, as the hydrogel does not affect the fluorescein derivative. When the 490 nm light was coupled into the hydrogel optical fiber through a multimode optical fiber, the immobilized fluorescence derivative was excited, and the fluorescence was transmitted back to the spectrometer. Analyzing the fluorescence intensity, we could acquire pH around the hydrogel optical fiber. The hydrogel optical fiber was tested in the pH range of 3.4 to 9.0, and the fluorescence intensity for each pH over time was recorded (Figure 4A). When pH increased, the fluorescence intensity increased, and the fluorescence intensity was linearly correlated with pH in the pH range of 5.4–7.8 (Figure 4D). The response time of the fluorescent hydrogel optical fiber to pH is ~1 min for each pH increase. Response speed was related to two factors: fiber diameter and ion strength. The finer fiber diameter and higher ion strength made a faster response. We also measured the diameter of the pH sensing area of the fluorescent hydrogel optical fiber (Figure S5). The diameter was almost unchanged with pH changes, which meant pH variation brought no volume changes and optical loss.

Repeatability and continuous monitoring of the pH sensor were studied. The reversible protolytic equilibrium of the fluorescein makes dynamic pH detection based on fluorescein possible. Figure 4B shows four pH up and down cycles and the corresponding fluorescence intensity changes. Approximately 1 min was needed to reach the protolytic equilibrium, and the fluorescence intensity could recover to the same in every cycle. The repeatability and stability of the pH sensor were good. Then, we changed pH consecutively and recorded the fluorescence intensity changes. Figure 4C showed that the fluorescence intensity closely followed the pH changes even if the pH changed randomly. Response time was ~1 min for each pH change. Meanwhile, there was no hysteresis for pH change in the range of 5.4 to 7.8. The pH sensor was stable under long-time illumination (Figure S6).

### 3.3. Difunctional Fluorescent Hydrogel Optical Fibers for Glucose Monitoring

**Scheme 1:**
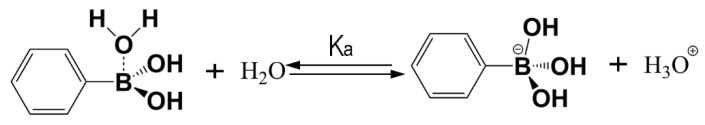
The equilibrium between phenylboronic acid and the conjugate phenylboronate in aqueous media.

PBA derivatives-based nonenzymatic glucose detection was studied extensively as cis-1,2-diols are prone to complex with PBA derivatives to form five-membered cyclic arylboronate esters [19,43]. Experimental observations clearly show that the corresponding arylboronate is the more reactive species in forming esters with neutral diols [56]. pH will influence the formation of arylboronate, as Scheme 1 shows. Tetrahedral anionic boronate, corresponding to the PBA derivatives, is formed and further complex with glucose when pH is greater than pKa. PBA derivatives functionalized hydrogels were also fabricated for glucose detection [41]. When glucose molecules diffuse into the hydrogel and complex (1:1) with PBA derivatives, the Donnan osmotic pressure increases and the volume of the hydrogel expands [57]. Glucose can be quantified by measuring the physical parameters related to the volume change in a well-designed sensor. A 3-APBA functionalized hydrogel optical fiber doped with QDs was fabricated for continuous glucose detection. The diameter changes of the functionalized hydrogel optical fiber in 0–10 mM glucose solution (0.1 M PBS, pH 7.4) were recorded. Approximately 25 min were needed to reach the equilibrium for the arylboronate ester formation. In comparison, there were few diameter changes for blank hydrogel (Figure S7). 

**Figure 5:**
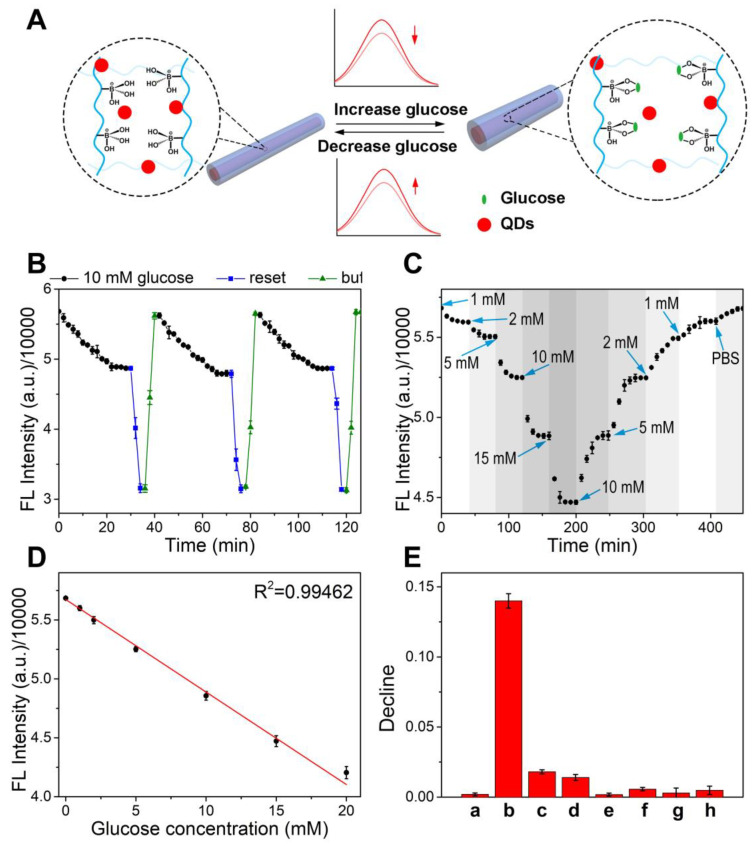
(**A**) Schematic diagram of volume change of the hydrogel and the fluorescence change caused by glucose concentration changes. (**B**) Time response of the sensor for going through three consecutive glucose additions, buffering, and sensor resetting cycles. The dots represent the fluorescence intensity recorded every 2 min. (**C**) Time response of the sensor for continuous glucose monitoring. The dots represent the fluorescence intensity recorded every 8 min. The moments when pH start to change are pointed out with arrows. (**D**) Linear detection for glucose of the sensor. (**E**) Percentage of fluorescence intensity decline; a–h: blank hydrogel, glucose, lactate (0.75 mM), fructose (8.1 μM), K^+^, Na^+^, Mg^2+^, Ca^2+^. Error bars in (**B**–**E**) are based on standard deviations (n = 3).

The QDs played as a fluorescence provider and converter, which converted the volume changes to fluorescence intensity changes. QDs themselves do not respond to glucose. The 490 nm light was coupled into the hydrogel optical fiber through the multimode optical fiber to excite the QDs, and the fluorescence was transmitted back to the spectrometer. As Figure 5A shows, the hydrogel fiber volume expanded gradually when the sensor was immersed in the glucose solution. The volume expansion decreased the RI and the density of the hydrogel fiber, and the optical loss increased correspondingly. The fluorescence intensity transmitted to the spectrometer decreased as the optical loss of the hydrogel fiber increased. On the other hand, the pKa of the arylboronic acid diester is typically 2–3 units smaller than the pKa of the corresponding arylboronic acid [58]. The local pH decrease due to the formation of the arylboronate ester would also waken the fluorescence intensity as QDs are pH sensitive (Figure S8).

The time response of the sensor in 10 mM glucose solution (PBS, pH 7.4) is shown in Figure 5B. Approximately 25 min were needed to reach the equilibrium, and the fluorescence intensity decreased by 14% as the glucose solution was replaced with acetate buffer (pH 6.2). The arylboronate ester transformed into phenylboronic acid, and glucose was released. The decrease of the Donnan osmotic pressure resulted in the shrinkage of the hydrogel fiber. Nevertheless, the fluorescence intensity did not increase as pH dominated the change in the fluorescence intensity. Then, the fluorescence intensity immediately recovered to the initial value when the acetate buffer was replaced with PBS (pH 7.4). Figure 5B shows three times of repetitions of adding glucose, buffering, and resetting. There was no hysteresis recorded during repetitions.

On account of the reversibility of complexation between glucose and PBA, the 3-APBA functionalized hydrogel fiber doped with QDs could be applied in continuous glucose monitoring [59]. Fluorescence intensity followed changes in glucose concentration ranging from 1 mM to 10 mM (Figure 5C). It took ~25 min to reach the equilibrium for each increase in glucose concentration. Fluorescence intensity changed linearly with glucose in the range of 0 to 20 mM (Figure 5D), which covered the physiological glucose concentration range (normal: 4.2–6.4; diabetic: 3.0–20.0). The decomplexation time was ~40 min for each glucose concentration decrease (Figure 5C). When the glucose concentration returned to the same level during the drop in the glucose concentration, fluorescence intensity recovered. No hysteresis occurred in dynamic changes. Thus, the sensor can be used for continuous glucose dynamic monitoring in the long term.

We also studied the influences of interferents on glucose detection of the sensor, including fructose, lactate, and some common ions (K^+^, Na^+^, Ca^2+^, Mg^2+^). The functionalized hydrogel fiber was immersed in the solution containing glucose, fructose, lactate (10 mM), K^+^, Na^+^, Ca2^+^, and Mg^2+^ (150 mM). Time responses of the sensor to glucose, fructose, and lactate are shown in Figure S9. The result showed a higher affinity of phenylboronic acid to lactate and fructose than glucose. They produced apparent interferences to glucose detection of 3-APBA-based sensors. However, concentrations of lactate in healthy resting people and fructose for healthy people were 0.36–0.75 mM and ~8.1 μM, respectively [60,61]. The interferences of lactate and fructose under normal concentrations are shown in Figure 5E. Thus, lactate and fructose interferences can be ignored in practical use. The functionalized hydrogel fiber would shrink when immersed in ion solution (150 mM) because of nonspecific charge interactions between the metal ions and hydrogel fiber matrix, resulting in decreased Donnan potential [41]. The fluorescence changed little as the shrinkage and ions brought almost no influence to QDs (Figure 5E).

pH will affect the glucose detection of 3-APBA functionalized hydrogel fiber as pH is the dominating factor in the arylboronate ester formation. QDs are also pH sensitive. Figure S10 shows the pH response of the sensor in the solution with 10 mM glucose. The hydrogel fiber diameter reduced with pH decrease, resulting in lower optical loss and collected fluorescence intensity enhancement. On the other hand, QDs’ fluorescence intensity declined due to a pH decrease. Under the comprehensive effects, QDs pH sensitivity dominated the fluorescence changes when pH was less than 7.4, and hydrogel expansion dominated the fluorescence changes when pH was greater than 7.4. When pH was fixed, the collected fluorescence intensity was only affected by glucose. The sensor showed different detection curves at different pH (Figure S11). Coupling a pH sensor into the 3-APBA functionalized hydrogel fiber to calibrate pH would enable it to monitor glucose in various pH conditions.

### 3.4. Difunctional Fluorescent Hydrogel Optical Fibers for Simultaneous Continuous pH and Glucose Monitoring

**Figure 6:**
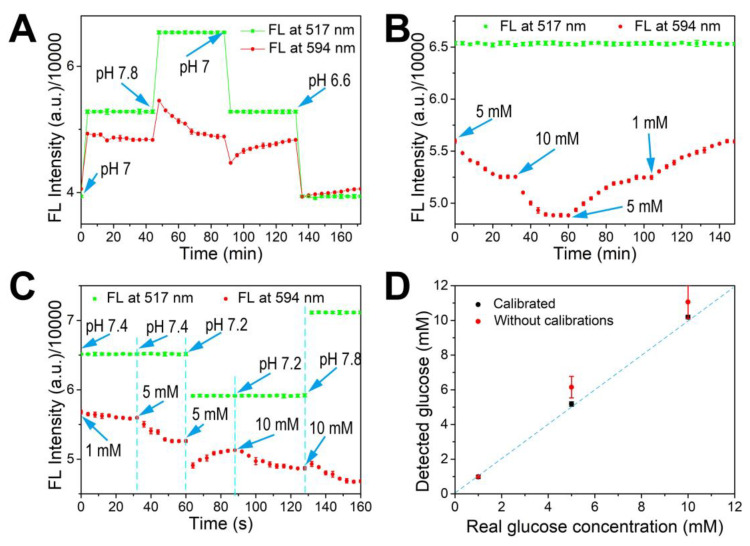
(**A**) Time response of fluorescence intensity at 517 nm and 594 nm when pH changed in the range of 6.6–7.8 with a fixed glucose concentration of 10 mM. The dots represent the fluorescence intensity recorded every 4 min, which is the same as (**B**,**C**). The moments when pH and glucose concentration start to change are pointed out with arrows, which are the same as (**B**,**C**). The solid line is for ease of understanding. (**B**) Time response of fluorescence intensity at 517 nm and 594 nm when glucose concentration changed in the range of 1–10 mM with fixed pH at 7.4. (**C**) Time response of fluorescence intensity at 517 nm and 594 for cross changes of pH and glucose concentration. The cyan vertical dashed lines play the same role as the arrows. (**D**) Deviation of glucose detection in (**C**). The blue dashed line indicates that the detected glucose concentration equals the actual glucose concentration. All error bars are based on standard deviations (n = 3).

Separate detection performances of the difunctional fluorescent hydrogel optical fiber were introduced above. The two-parameter simultaneous detection of the sensor was discussed next. The 490 nm light was coupled into the hydrogel fiber through the multimode optical fiber. The dual-emission fluorescence was collected by the spectrometer. Firstly, the hydrogel fiber was immersed in the solution with a fixed glucose concentration at 10 mM. As pH decreased from 7.8 to 6.6, fluorescence intensities at 517 nm declined, and fluorescence intensities at 594 nm climbed up and then declined, which was in accordance with the above discussions (Figure S12). Continuous pH monitoring was performed in the pH range of 6.6 to 7.8, with 10 mM glucose (Figure 6A). The red fluorescence for glucose detection was influenced by pH variations though the glucose concentration was fixed. The red fluorescence experienced a rapid enhancement and a slow decline when pH increased. This is because the PBA-hydrogel has a faster response to pH than glucose. Then, pH was fixed at 7.4, and the fluorescence spectra were recorded when glucose concentration changed from 1 to 10 Mm (Figure S13). The glucose concentration variation brought no influences on green fluorescence. Figure 6B shows the time response of the sensor for continuous glucose monitoring. The fluorescence intensity at 594 nm declined as glucose concentration increased without changes in green fluorescence. The glucose detection curves in different pH were shown in Figure S11. pH detection was immune to glucose concentration changes. After pH calibrating, glucose detection could be realized by contrasting the collected fluorescence intensity with the premeasured detection curve. 

The sensor was also tested in cross changes of pH and glucose concentration. The hydrogel fiber was first immersed in the initial PBS solution (pH 7.4) with 1 mM glucose. Then, the hydrogel fiber went through 5 mM glucose (pH 7.4), 5 mM glucose (pH 7.2), 10 mM glucose (pH 7.2), and 10 mM glucose (pH 7.8). Time responses are shown in Figure 6C. Glucose concentration was calibrated by contrasting the measured result with detection curves measured before. Figure 6D shows the detected glucose in comparison with the actual glucose concentration. The deviation was less than 3%. Without calibrated results, pH changes would bring large deviations (>10%) and significant fluctuations in glucose monitoring. Thus, pH calibration is necessary for pH-sensitive glucose sensors. The sensor performed well in simultaneous continuous glucose and pH monitoring when pH was calibrated. Furthermore, we have compared this work with the reported work (Table 1 and Table 2).

## 4. Conclusions

We developed a difunctional fluorescent hydrogel optical fiber for simultaneous continuous glucose and pH monitoring. The sensor has advantages over the reported sensing method in continuous monitoring and self-calibrating. The hydrogel fiber was segmentally functionalized with fluorescein derivative and quantum dots/3-APBA. The sensitive agent fluorescein derivative and quantum dots/3-APBA correspond to pH and glucose detection, respectively. Two fluorophores were excited when coupling 490 nm light into the hydrogel fiber through the multimode fiber, and the fluorescence was transmitted back to the spectrometer. Moreover, the two different emissions (517 nm of fluorescein derivative and 594 nm of QDs) enable the sensor’s simultaneous detection of pH and glucose. We can acquire the glucose concentration and pH by analyzing the fluorescence intensity. On the other hand, pH monitoring is a calibration for glucose monitoring to improve detection accuracy. The sensor can continuously monitor glucose in 0–20 mM and pH in 5.4–7.8. In addition, benefiting from the reversibility of the two sensing reactions, the difunctional fluorescent hydrogel optical fiber can be used as a dynamic sensor. The simultaneous continuous simultaneous monitoring experiment indicates that the sensor has a high tolerance even if pH and glucose concentration change randomly. Furthermore, there is very little interference in practical use. The sensor can be implanted due to the biocompatible and flexible hydrogel matrix. If the PBA derivative with low pKa (<7) is introduced to the hydrogel fiber, the sensor can detect glucose in acidic conditions and be used in wearable sensors for sweat glucose detection.

## Figures and Tables

**Table 1:** Comparison of the proposed sensor with other glucose detection methods.

Probe	Type	Linear Range (mM)	Continuous Monitoring	References
CQDs-GOD/CA	Enzyme	0.01–0.2	No	[62]
PIGC	Enzyme	0.52–6.47	Yes	[63]
PMIGC	Enzyme	0.46–38.89	No	[64]
CdTe QDs/3-APB	Enzyme free	0–20	Yes	This work

**Table 2:** Comparison of the proposed sensor with other pH detection methods.

Fluorescence Probe	Response Range (pH)	Repeatability	References
DLF and PtTPTBP	3.79–9.55	Not mentioned	[30]
DAOTA and TDI	4.6–7.6	Not mentioned	[65]
DAOTA and DMQA	3.8–6.8	Good	[66]
Fluorescein derivative	5.4–7.8	Good	This work

## Data Availability

The data presented in this study are available on request from the corresponding author. The data are not publicly available due to privacy restrictions.

## Supplementary Materials

The following supporting information can be downloaded at: https://www.mdpi.com/article/10.3390/bios13020287/s1, Figure S1. Mechanical measurements of the hydrogel the optical fiber; Figure S2. Excitation independence of CdTe QDs; Figure S3. Absorption spectra of fluorescein derivative in different pH; Figure S4. Excitation independence of fluorescein derivative; Figure S5. Diameter changes of the hydrogel fiber in different pH. Error bars are based on standard deviations (n = 3); Figure S6. The pH sensor was stable under long time illumination; Figure S7. Diameter of the hydrogel fiber changes with glucose concentration. Error bars are based on standard deviations (n = 3); Figure S8. pH response of QDs from 3–10. Error bars are based on standard deviations (n = 3); Figure S9. Time response of the sensor in glucose, fructose and lactate. The dots represent the fluorescence intensity recorded every 5 minutes. Error bars are based on standard deviations (n = 3); Figure S10. pH response of the sensor for glucose detection; Figure S11. Detction curves of the sensor in different pH. Error bars are based on standard deviations (n = 3); Figure S12. Fluorescence spectra of the sensor at pH 6.6, 7.4, 7.8 with 10 mM glucose; Figure S13. Fluorescence spectra of the sensor at pH 7.4 with 1 and 10 mM glucose; Scheme S1. Synthesis of fluorescein derivative; Scheme S2. Synthetic scheme of PAM-co-PEG-3-APBA; Scheme S3. Synthetic scheme of amino hydrogel; Scheme S4. Synthetic scheme of fluorescein derivative functionalized hydrogel.
