# Supplementary material for: Difunctional Hydrogel Optical Fiber Fluorescence Sensor for Continuous and Simultaneous Monitoring of Glucose and pH
Source: Biosensors (Basel). 2023 Feb 17;13(2):287. doi: 10.3390/bios13020287 (PMC9954304; doi:10.3390/bios13020287)
Supplement: Supplementary file 1 [file biosensors-13-00287-s001.zip › biosensors-2204233-supplementary.pdf]

Difunctional hydrogel optical fiber fluorescence sensor for continuous and simultaneous monitoring of glucose and pH

Yangjie Li, Site Luo, Yongqiang Gui, Xin Wang, Ziyuan Tian, Haihu Yu*
National Engineering Research Center of Fiber Optic Sensing Technology and Networks, Wuhan University of Technology, Wuhan 430070, China
*E-mail: hhyu@whut.edu.cn

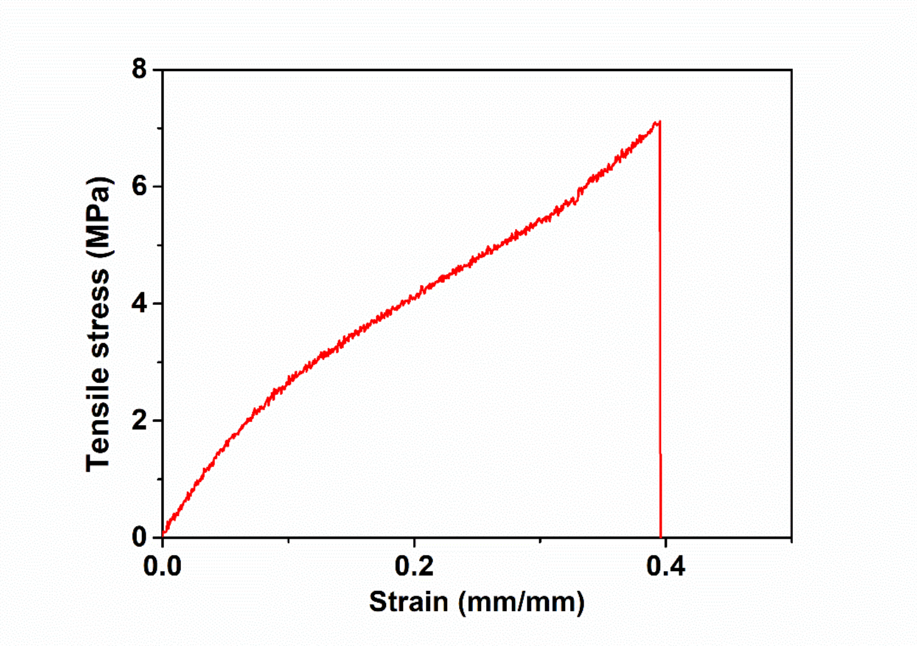

Figure S1. Mechanical measurements of the hydrogel the optical fiber.

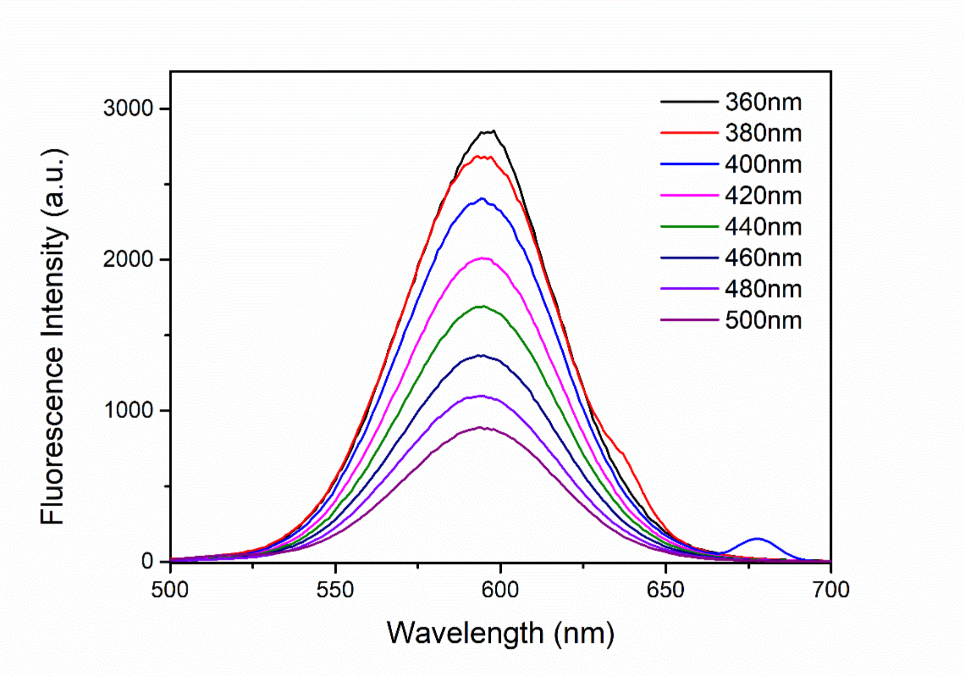

Figure S2. Excitation independence of CdTe QDs.

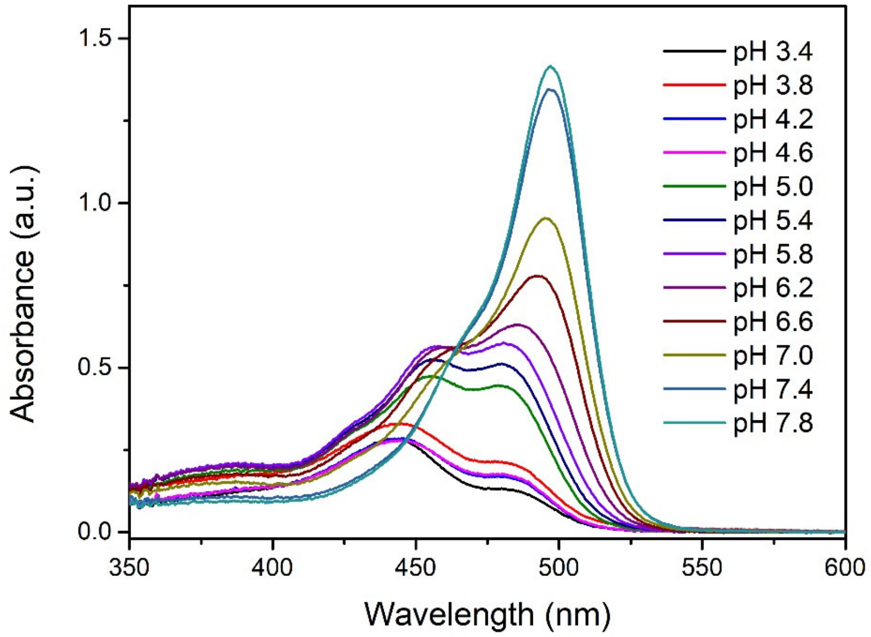

Figure S3. Absorption spectra of fluorescein derivative in different pH.

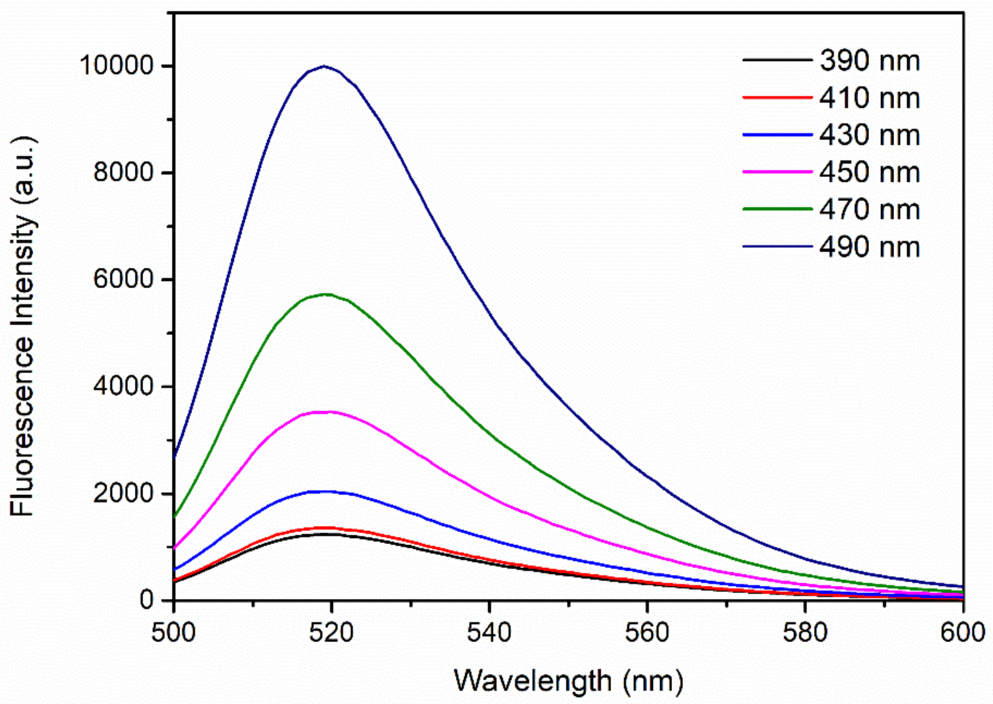

Figure S4. Excitation independence of fluorescein derivative.

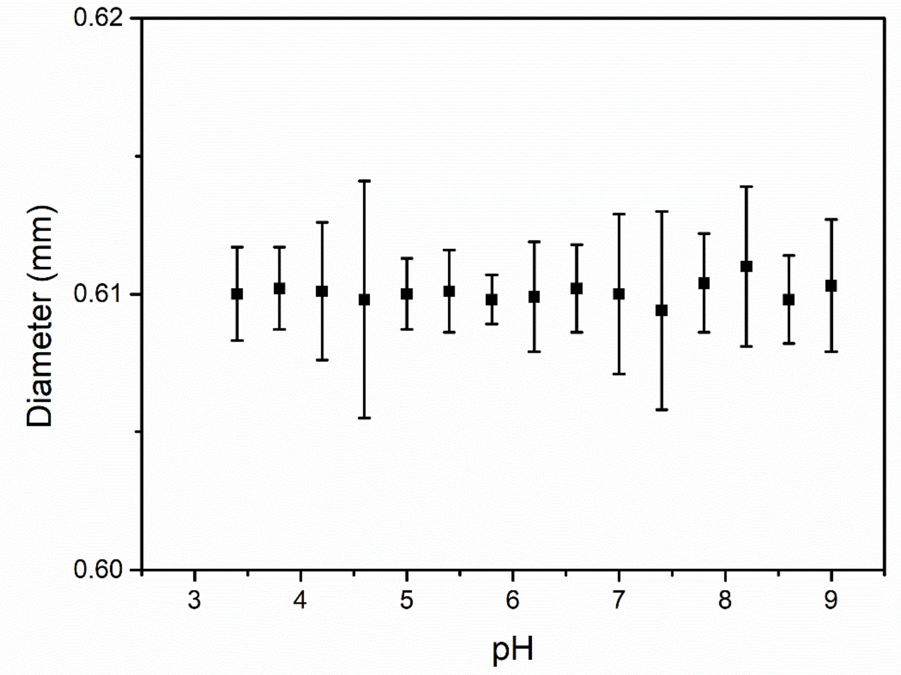

Figure S5. Diameter changes of the hydrogel fiber in different pH. Error bars are based on standard deviations (n = 3).

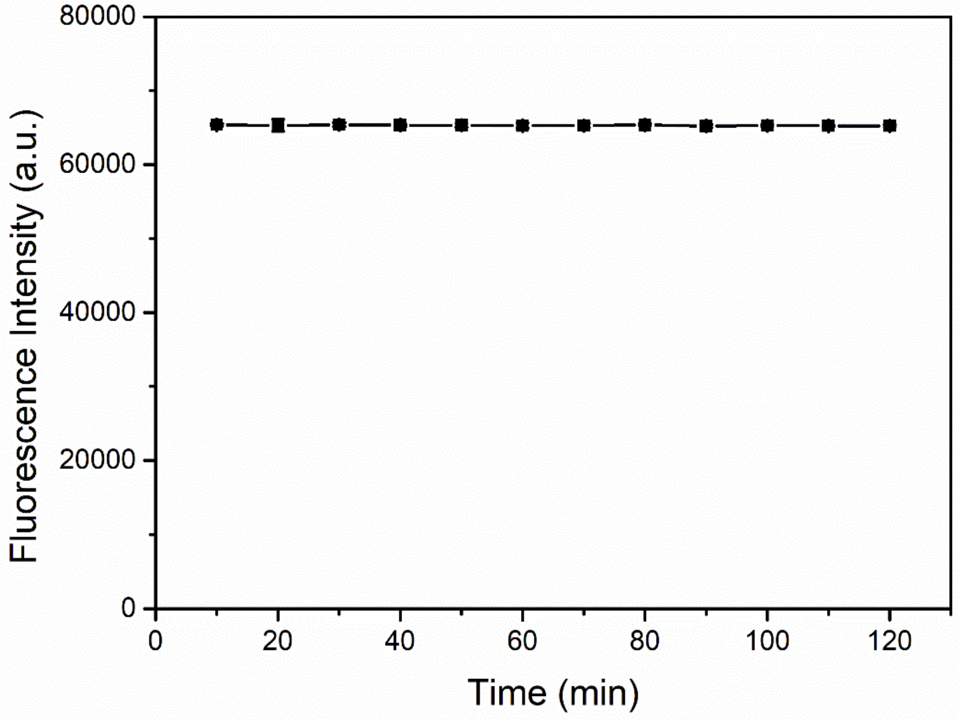

Figure S6. The pH sensor was stable under long time illumination.

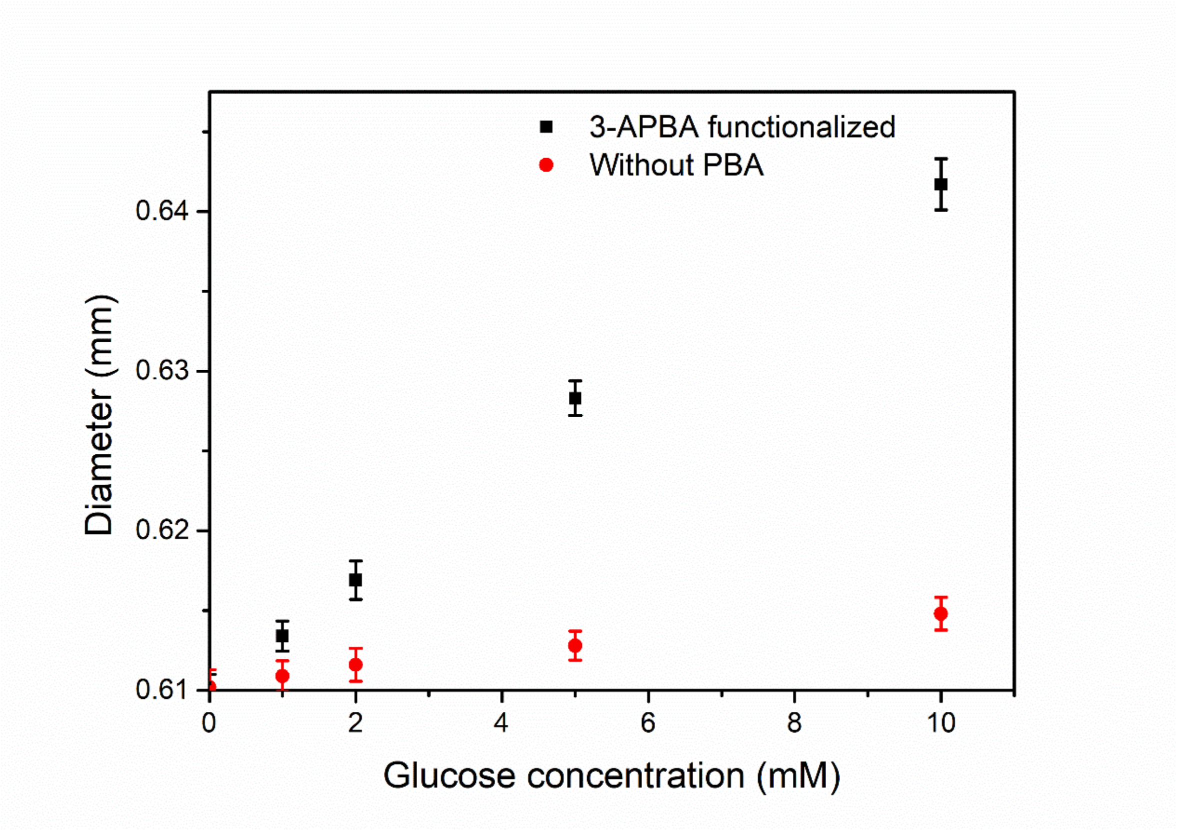

Figure S7. Diameter of the hydrogel fiber changes with glucose concentration. Error bars are based on standard deviations (n = 3).

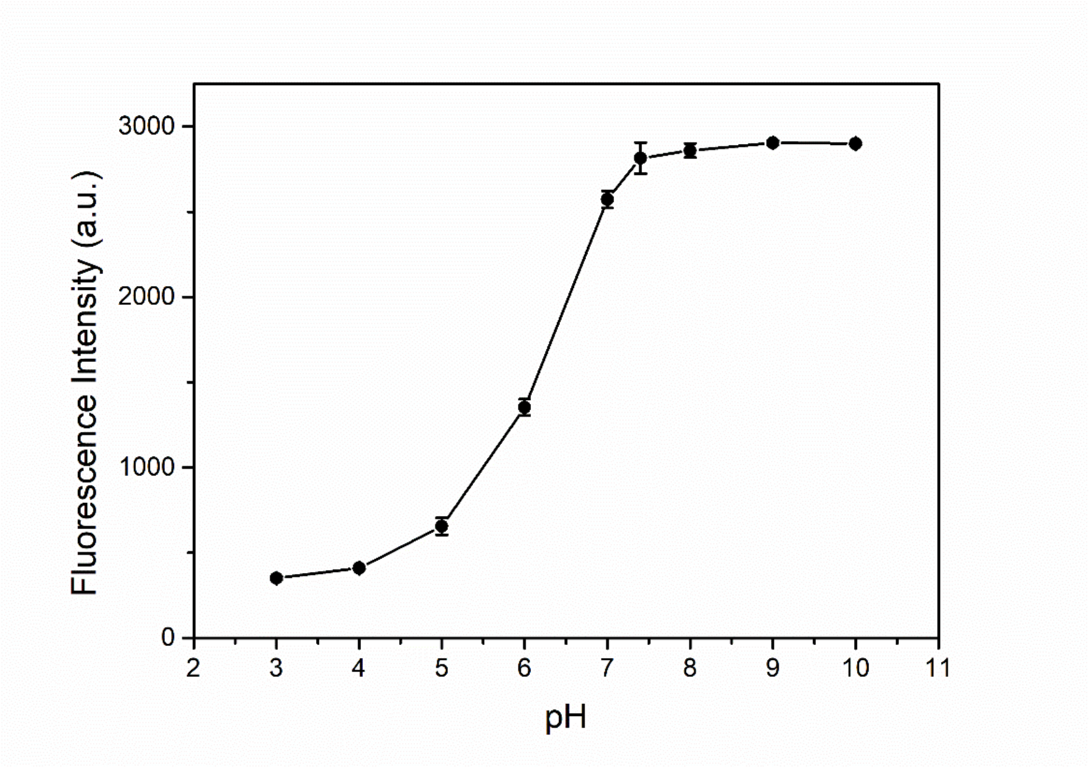

Figure S8. pH response of QDs from 3-10. Error bars are based on standard deviations (n = 3).

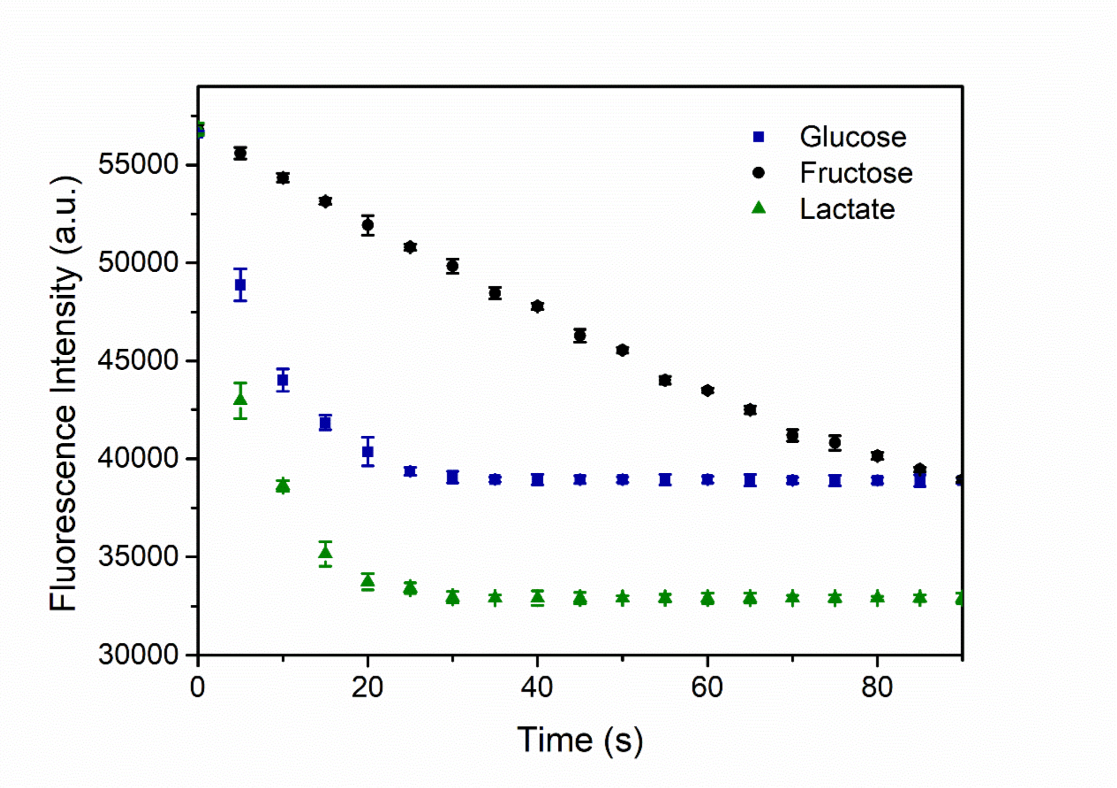

Figure S9. Time response of the sensor in glucose, fructose and lactate. The dots represent the fluorescence intensity recorded every 5 minutes. Error bars are based on standard deviations (n = 3).

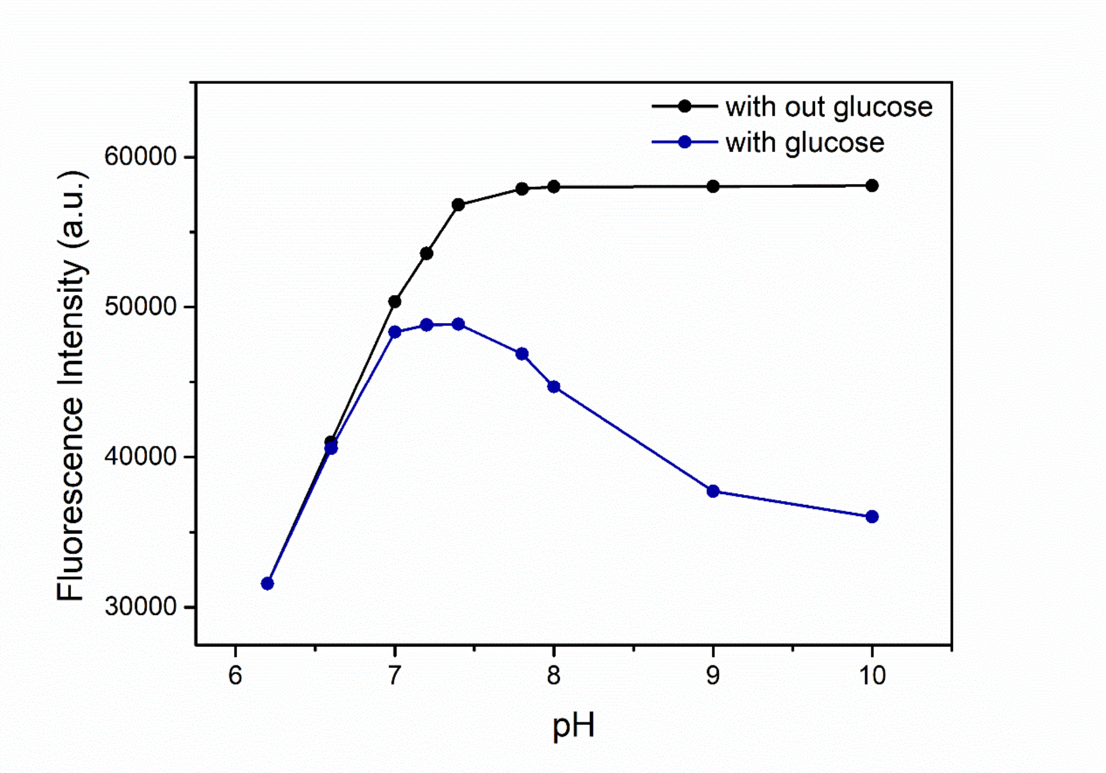

Figure S10. pH response of the sensor for glucose detection.

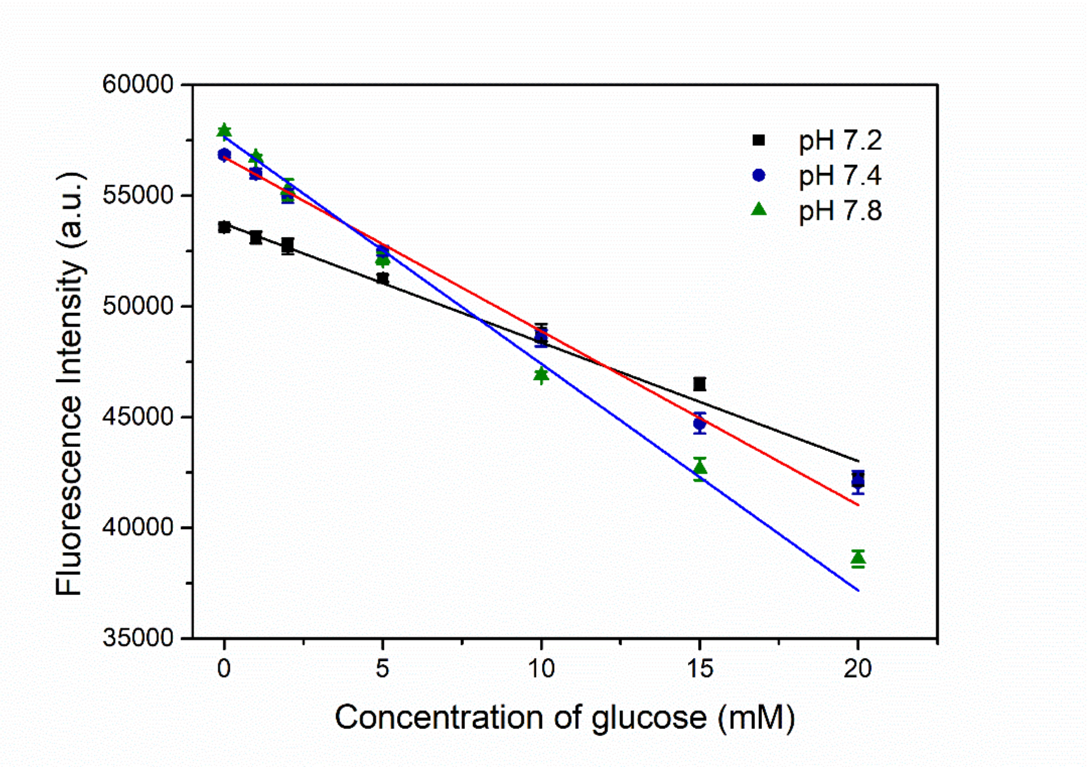

Figure S11. Detction curves of the sensor in different pH. Error bars are based on standard deviations (n = 3).

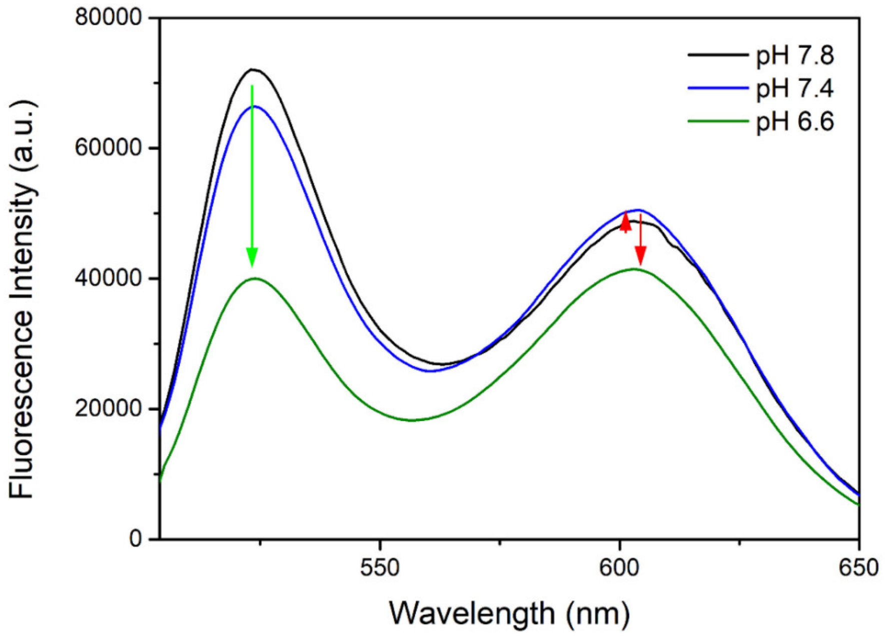

Figure S12. Fluorescence spectra of the sensor at pH 6.6, 7.4, 7.8 with 10 mM glucose.

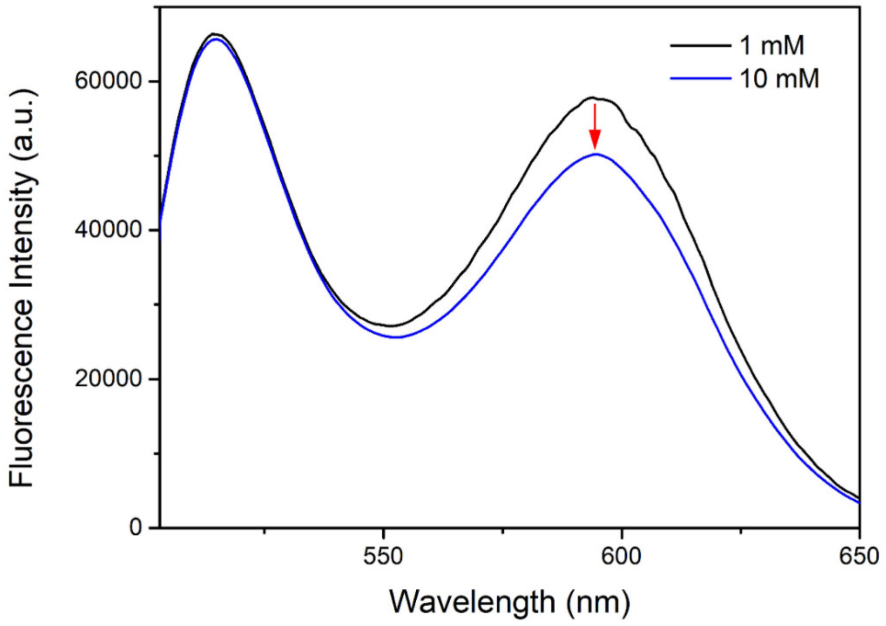

Figure S13. Fluorescence spectra of the sensor at pH 7.4 with 1 and 10 mM glucose.

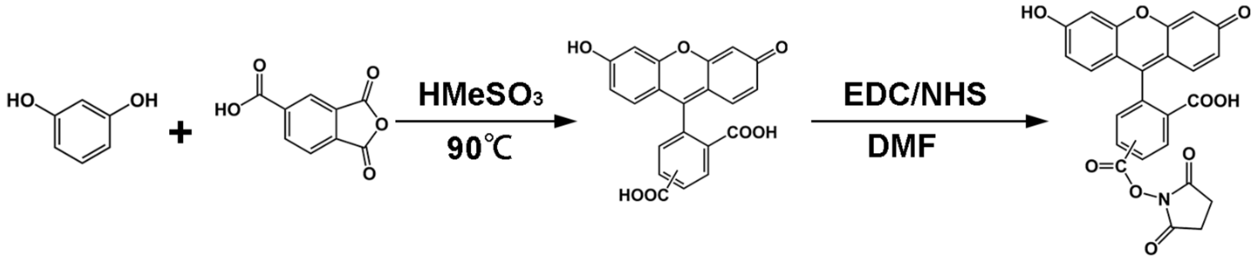

Scheme S1. Synthesis of fluorescein derivative.

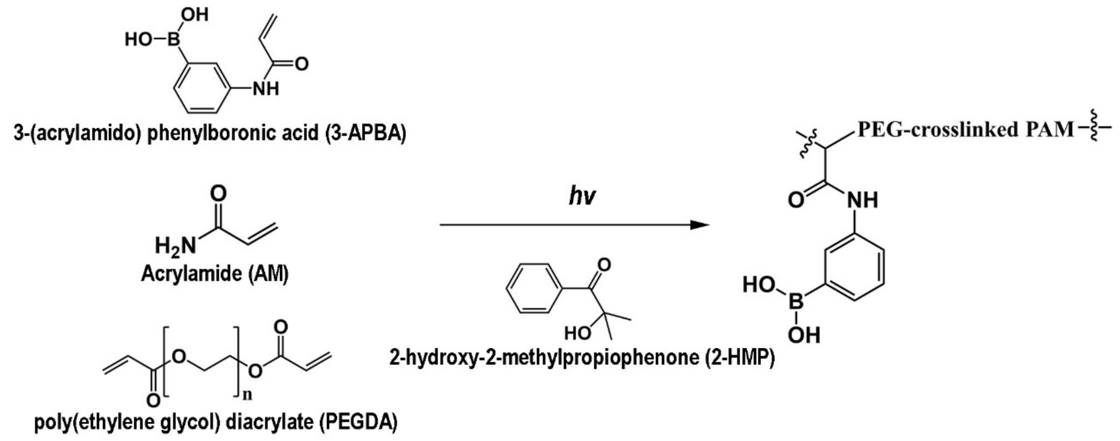

Scheme S2. Synthetic scheme of PAM-co-PEG-3-APBA.

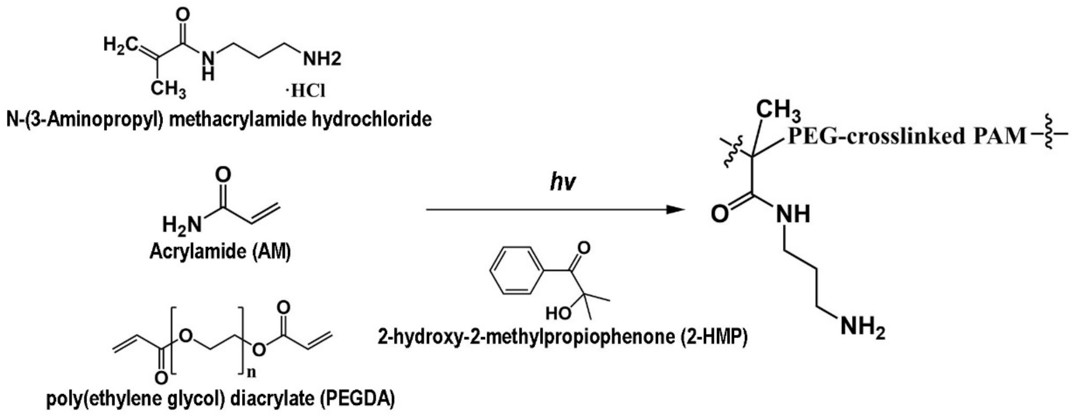

Scheme S3. Synthetic scheme of amino hydrogel.

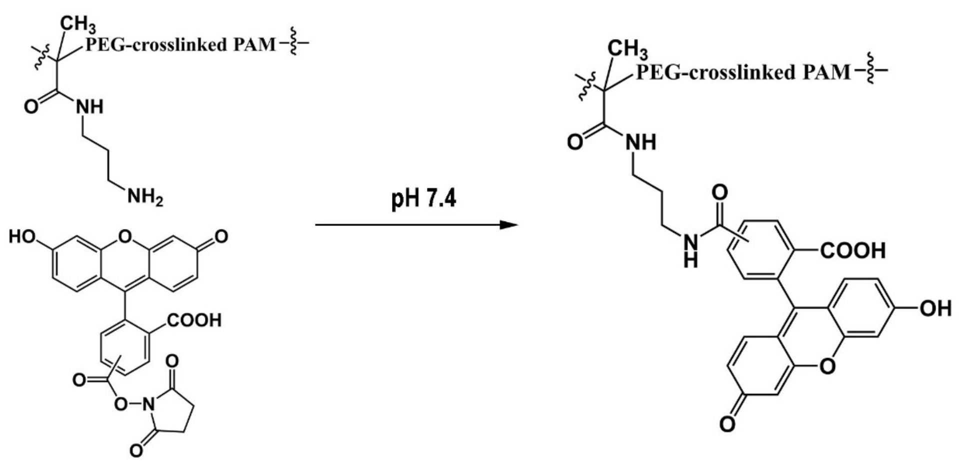

Scheme S4. Synthetic scheme of fluorescein derivative functionalized hydrogel.
